# The Association Between Primary Sclerosing Cholangitis and Microscopic Colitis: A Systematic Review

**DOI:** 10.7759/cureus.75587

**Published:** 2024-12-12

**Authors:** Hasan Al-Obaidi, Mustafa Al-Obaidi, Pratiksha Moliya, Hussein Harb, Yusuf Nawras, Nooraldin Merza

**Affiliations:** 1 Internal Medicine, Jamaica Hospital Medical Center, New York, USA; 2 Internal Medicine, Lund University, Lund, SWE; 3 Graduate Medical Education, Shri MP Shah Medical College, Jamnagar, IND; 4 Basic Sciences, Ross University School of Medicine, Bridgetown, BRB; 5 Medicine, The University of Toledo College of Medicine and Life Sciences, Toledo, USA; 6 Internal Medicine, Wayne State University School of Medicine, Dearborn, USA

**Keywords:** autoimmune disease, collagenous colitis, gut dysbiosis, inflammatory bowel disease, lymphocytic colitis, microscopic colitis, primary sclerosing cholangitis (psc), ulcerative colıtıs

## Abstract

The association between primary sclerosing cholangitis (PSC) and microscopic colitis (MC) has been explored in limited studies, suggesting potential shared pathophysiological mechanisms. This systematic review aimed to investigate this relationship by analyzing studies identified through comprehensive searches in PubMed, Embase, and the Cochrane Library. Two studies met the inclusion criteria: a case series of 12 patients and a case report, collectively analyzing 13 cases. The case series revealed that 75% of MC diagnoses occurred after PSC, with many cases being asymptomatic, suggesting potential underdiagnosis. The case report described a patient with collagenous colitis who developed severe PSC complications, underscoring the bidirectional relationship and clinical impact of these conditions. Both studies highlighted immune dysregulation, genetic predisposition (HLA-DR3, HLA-DRw52a), and alterations in gut flora as shared mechanisms. These findings emphasize the importance of increased clinical vigilance, early diagnosis, and management of MC in PSC patients. Further research is needed to validate these associations, evaluate routine screening, and explore therapeutic approaches.

## Introduction and background

Primary sclerosing cholangitis (PSC) is a chronic, progressive cholestatic liver disease marked by inflammation and fibrosis of the intrahepatic and extrahepatic bile ducts [1]. This causes biliary strictures and cholestasis [2]. The pathogenesis of PSC is unknown [3,4]; however, it is thought to be a complex disease, including genetic predisposition [4], environmental factors, and immunological dysregulation [5,6]. PSC is frequently associated with inflammatory bowel disease (IBD) [7,8], including ulcerative colitis (UC) [9], with up to 80% of PSC patients having concurrent IBD [10]. PSC frequently begins slowly with fatigue, pruritus, and jaundice, and can proceed to cirrhosis, liver failure, and cholangiocarcinoma [11-14].

MC is a chronic inflammatory disorder of the colon characterized by watery diarrhea and normal endoscopic appearance [15-16]. The pathophysiology of MC is also unknown [17]; however, it is thought to include dysregulated immune responses [18], altered gut flora [19,20], and genetic susceptibility [21]. The histological study of colonic samples reveals typical inflammatory alterations, serving as the basis for the diagnosis of MC. The two subtypes of MC are collagenous colitis (CC) [22] and lymphocytic colitis (LC) [23], which differ by the presence of a thicker subepithelial collagen layer in CC [24].

While PSC and MC are generally accepted as different diseases, the literature provides limited evidence regarding MC patients with concurrent PSC [25]. Sehgal et al. suggest that patients with PSC and unexplained diarrhea, as well as those with MC and abnormal liver function tests, should be managed with the possibility of concurrent MC and PSC [26]. This correlation points to the common pathogenetic processes, such as abnormal immunological responses and gut dysbiosis, which may contribute to the development and progression of both disorders [27].

Understanding the nature and scope of the relationship between PSC and MC is critical for a variety of reasons. First, it may shed light on the underlying mechanisms that drive both diseases, potentially leading to the identification of new treatment targets. Second, it may assist clinicians in identifying patients who are more likely to acquire either ailment, allowing for earlier diagnosis and intervention. 

The purpose of this systematic review is to describe current data on the relationship between PSC and CC, including the epidemiology, etiology, clinical symptoms, diagnosis, and therapy of this complicated illness. We'll also discuss how this association might affect clinical practice and future research.

## Review

A comprehensive literature search was conducted in the PubMed, Embase, and Cochrane Library databases, spanning inception to May 2024, to investigate the relationship between primary sclerosing cholangitis (PSC) and microscopic colitis (MC). Keywords included "primary sclerosing cholangitis," "microscopic colitis," "lymphocytic colitis," "collagenous colitis," "association," "co-occurrence," and "prevalence." Additional studies were identified by manually screening references in relevant publications. Studies were included if they provided original data on patients diagnosed with both PSC and MC, explicitly discussed associations between lymphocytic colitis (LC) or collagenous colitis (CC) and PSC, and included sufficient clinical, diagnostic, or therapeutic information, all published in English. Exclusion criteria ruled out studies that were review articles, editorials, abstracts, or lacking original data, as well as studies focusing solely on either condition or those published in non-English languages.

Data from eligible studies were systematically extracted using a standardized form to capture research design, sample size, patient demographics, MC subtypes, PSC diagnostic methods, symptomatology, treatment responses, and clinical outcomes. Quality assessment was performed using the Joanna Briggs Institute's Critical Appraisal Checklist for Case Reports (https://jbi.global/critical-appraisal-tools), evaluating relevance, clarity, theoretical contributions, and consistency. Due to the limited number of studies and heterogeneity in study design, formal meta-analysis was not feasible. Instead, descriptive analysis and narrative synthesis were conducted, with results presented in tabular and graphical formats.

**Figure 1:**
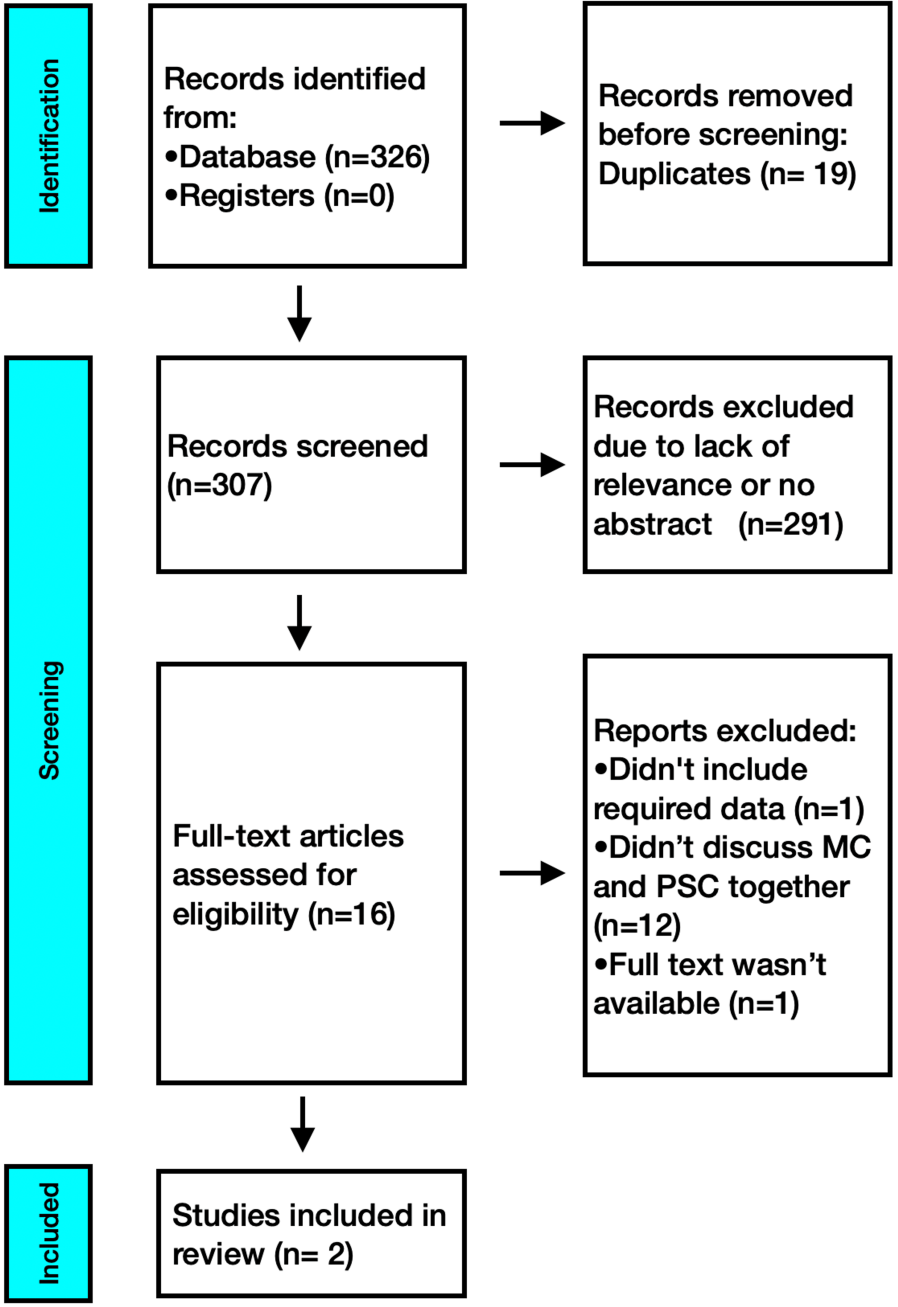
Preferred Reporting Items for Systematic Reviews and Meta-Analyses (PRISMA) flow diagram illustrating the study selection process for the systematic review of the association between primary sclerosing cholangitis (PSC) and microscopic colitis (MC)

The search initially yielded 326 studies, which were reduced to 307 after duplicates were removed. Of these, 291 were excluded following title and abstract review due to irrelevance, lack of discussion on combined PSC and MC, or absence of full text. Sixteen publications underwent full-text review, but 14 were excluded for not meeting eligibility criteria, leaving two studies for inclusion (Figure 1). These studies consisted of a single case report and a retrospective case series, collectively analyzing 13 cases. The retrospective study by Sehgal et al. examined 12 patients with both PSC and MC, including 7 with LC and 5 with CC, revealing that 75% of cases involved MC diagnoses occurring after PSC. This high proportion suggests potential underdiagnosis of MC in PSC populations, with asymptomatic cases identified incidentally [26].

The case report by Philips et al. described a 91-year-old female with collagenous colitis (CC) who later developed primary sclerosing cholangitis (PSC), highlighting the possible bidirectional relationship between the conditions (Tables 1, 2) [27]. Initially, the patient presented with persistent diarrhea, a negative stool sample, and an unremarkable colonoscopy, but histology revealed thickened submucosal collagen bands in the proximal and distal colon, confirming CC. Her symptoms resolved with a three-month course of budesonide. However, nine months later, she developed severe pruritus and cholestatic liver dysfunction (bilirubin 15, alanine aminotransferase (ALT) 101 IU/L, aspartate aminotransferase (AST) 35 IU/L, alkaline phosphatase (ALP) 1209 IU/L, gamma-glutamyl transferase (GGT) 1175 IU/L). Endoscopic retrograde cholangiopancreatography (ERCP) demonstrated a ragged common bile duct with irregular intrahepatic bile ducts, characteristic of PSC, and liver histology confirmed the diagnosis, ruling out immunoglobulin Ig4 (IgG4)-related disease. Despite biliary decompression, her condition deteriorated, and she passed away within two months. This case underscores the severe complications that can arise in PSC such as cholangitis, cirrhosis, and cholangiocarcinoma despite initial management of MC [27].

**Table 1:** Baseline characteristics of included studies

Characteristic	Philips et al. [27]	Sehgal et al. [26]
Study Design	Case report	Retrospective case series
Sample Size	1	12
Patient Demographics	91-year-old female	8 male, 4 female (median age 54.4 years, range 22.6-75.3 years)
MC Type	CC	7 LC, 5 CC
PSC Diagnosis	After MC	9 after MC, 3 before/concurrent with MC
MC Symptoms	Diarrhea	6 symptomatic, 6 asymptomatic
MC Treatment	Budesonide	5 treated (budesonide, prednisone, bismuth subsalicylate), 6 untreated
MC Treatment Response	Complete resolution	Complete resolution in treated patients
PSC Complications	Death	Cholangitis (5), strictures (7), cirrhosis (4), cholangiocarcinoma (2), death (1)
Other		1 with pre-existing UC, 1 with chronic colitis transformed to MC

**Table 2:** Joanna Briggs Institute (JBI) critical appraisal checklist for case reports: assessment of included studies

Criterion	Philips et al. [27]	Sehgal et al. [26]	Comments
Importance to practice/research	Yes	Yes	Both studies address a relevant clinical question.
Patient situation demonstrating importance	Yes	Yes	Both present clinical scenarios relevant to practice/research.
Clear definition of experience and importance	Yes	Yes	The focus of both studies is well-defined.
Uniqueness or unusualness of experience	Yes	Somewhat	Philips et al. is unique; Sehgal et al. highlight an unusual finding.
Sufficient detail for assessing clinical significance	Yes	Yes	Both provide enough clinical information.
Use of theoretical framework or previous research	Yes	Yes	Both studies reference relevant literature and theories.
Sufficient discussion of relevant literature	Yes	Yes	Both discuss relevant literature, but Sehgal et al. could be more in-depth.
Contribution to knowledge/understanding	Yes	Yes	Both studies add to the knowledge base.
Conclusions consistent with evidence	Yes	Yes	Conclusions in both studies are supported by the presented evidence.

Both studies provide insights into the potential shared pathophysiological mechanisms underlying PSC and MC. Common features include immune dysregulation and genetic predisposition. Evidence shows that both conditions are associated with human leukocyte antigen (HLA) class II alleles, such as HLA-DR3 and HLA-DRw52a, which are linked to autoimmune diseases [5]. Alterations in gut flora, common in PSC due to bile acid dysregulation, may also play a role in MC pathogenesis [26]. While the retrospective study identified no cases of dysplasia or malignancy among patients with MC and PSC, the severe complications of PSC, such as cholangitis, strictures, cirrhosis, and cholangiocarcinoma, were prominent in these patients [26,27].

Previous studies, such as Giraldo et al., support the autoimmune basis of this association by demonstrating a higher prevalence of autoimmune disorders (ADs) in MC patients, with 28.9% of MC cases linked to autoimmune comorbidity, including CC [28]. Similarly, a cohort study by Fedor et al. found that among 103 patients with biopsy-confirmed MC, autoimmune conditions were present in 40% of CC cases and 36% of LC cases, though the study did not specifically assess PSC [25]. These findings reinforce the likelihood of a broader autoimmune predisposition connecting PSC and MC.

Despite its contributions, this review has limitations. The small number of included studies and their retrospective design limit the generalizability of findings. The reliance on case reports and case series increases the risk of selection and recall bias. Furthermore, heterogeneity in diagnostic criteria for MC across studies could contribute to variability in reported results. Future studies should address these limitations by employing large-scale, prospective designs with standardized diagnostic protocols.

From a clinical perspective, the findings emphasize the need for increased vigilance in PSC patients for potential coexisting MC. Screening for MC, particularly in PSC patients presenting with unexplained diarrhea or abnormal liver function tests, could lead to earlier diagnosis and improved outcomes. Philips et al. highlighted the importance of effective treatment of MC symptoms, such as the use of budesonide for CC, which may enhance quality of life despite the progression of PSC [27]. Additionally, Sehgal et al. demonstrated the incidental detection of asymptomatic MC in PSC patients, underscoring the potential for undiagnosed cases in this population [26].

In conclusion, this review provides evidence of a possible association between PSC and MC, supported by overlapping immunological and genetic factors. The findings highlight the importance of awareness, early diagnosis, and management of MC in PSC patients. Further research is warranted to confirm these associations, explore shared mechanisms, and evaluate the efficacy of routine MC screening and treatment in PSC populations. Large-scale prospective studies could elucidate whether systematic screening for MC in asymptomatic PSC patients is justified and identify new therapeutic targets for both conditions.

## Conclusions

In conclusion, this systematic review highlights the emerging evidence for a potential association between primary sclerosing cholangitis (PSC) and microscopic colitis (MC). Although data are currently limited, the findings underscore the importance of increased awareness and consideration of MC in PSC patients, especially those presenting with unexplained diarrhea or abnormal liver function tests. Early identification and targeted treatment of MC could significantly improve patient outcomes and quality of life, particularly in the context of managing PSC-related complications.

Further research is needed to validate these observations through larger, prospective studies and to explore the underlying mechanisms connecting these two conditions. Such studies could offer valuable insights into shared pathophysiological pathways and potential therapeutic targets. Additionally, future investigations should aim to determine whether routine screening for MC in asymptomatic PSC patients is warranted and assess the long-term impact of early MC diagnosis and treatment on the overall disease trajectory. By advancing our understanding of the relationship between PSC and MC, clinicians may be better equipped to provide comprehensive care for affected individuals.
